# First Report of the HEV Seroprevalence and the Risk Factor Assessment in the West Bank, Palestine, during the Period of 2015–2017

**DOI:** 10.1155/2022/4935811

**Published:** 2022-02-08

**Authors:** Kamal Dumaidi, Alaa M. Abudamous, Rasmi Abu-Helu, Hanan Al-Jawabreh, Yazan Dumaidi, Amer Al-Jawabreh

**Affiliations:** ^1^Department of Medical Laboratory Sciences, Faculty of Allied Health Sciences, Arab American University Palestine, Jenin, State of Palestine; ^2^Al-Quds Primary Health Care, Palestinian Ministry of Health, Ramallah, State of Palestine; ^3^Department of Medical Laboratory Sciences, Faculty of Health Professions, Al-Quds University, Jerusalem, State of Palestine; ^4^Leishmaniases Research Unit, Jericho, State of Palestine; ^5^Faculty of Medicine and Health Sciences, An Najah National University, Nablus, State of Palestine

## Abstract

Hepatitis E virus is emerging viral hepatitis with hyperendemicity in many countries. Data on the burden of disease is not available in Palestine. This study aims to determine the seroprevalence and the risk factors of the HEV among the general population of the West Bank, Palestine. In this cross-sectional study, a total of 432 sera samples from 40 localities in the eleven districts of the West Bank and Jerusalem, Palestine, during the period of March 2015 to March 2017, were tested for HEV-IgG. A structured questionnaire was used to collect data of the participants' demographics and disease risk factors. The overall seroprevalence was 3.7%. Level of education was significantly inversely associated with HEV seropositivity (*P*=0.04). Purely spatial analysis did not detect any significant cluster related to the distribution of HEV-IgG cases; however, living in the southern West Bank is shown to be significantly associated with HEV. Age was also associated with HEV seropositivity. The young (<19 years) and adults (>40 years) had the highest prevalence, compared to those between 20 to 39 years old (*P*=0.12). Furthermore, males and those in contact with animals were associated with HEV seropositivity (*P*=0.1 and 0.3, respectively). In conclusion, the seroprevalence of HEV IgG in the West Bank, Palestine is low. Several well-investigated risk factors cannot be supported by our results due to the small number of the positive HEV-IgG samples. Finally, this study is useful for providing a first look into the seroepidemiology of HEV in Palestine.

## 1. Introduction

Hepatitis E virus (HEV) is a small, nonenveloped, positive sense, single-stranded RNA (ssRNA) virus that belongs to the genus *Hepevirus* of the family *Hepeviridae* [1]. The infection is usually asymptomatic or self-limited, but it may progress to acute liver disease with symptoms ranging from subclinical to fulminant hepatitis, especially in pregnant women, and to chronic infection that has also been reported in immunocompromised patients. The most common clinical presentations of acute HEV infection are jaundice, fatigue, anorexia, abdominal discomfort, fever, dark urine, hepatomegaly, and splenomegaly [2–4].

HEV is considered as an important public health concern with the WHO annually reporting 20 million infections, 3.3 million symptomatic cases, and a round of 44000 deaths [5]. HEV is a food-borne disease transmitted mainly through the consumption of contaminated water and food in a poorly hygienic atmosphere in developing countries and via the consumption of undercooked pork as a food-borne zoonosis in industrialized countries [6, 7]. Furthermore, transfusion of blood and blood products has also been reported as a mode of transmission of HEV infection [8] as a result of transient HEV viremia among asymptomatic infected blood donors. The wide variation of prevalence of HEV-IgG among blood donors (5.7%–86.4%) has been reported [3, 9–12].

To date, eight HEV genotypes have been identified [13]. Genotypes 1 to 4 are reported to cause human infection [11]. Genotypes 1 and 2 are endemic to Southeast Asia, Africa, and South America and typically spread via contaminated water [14–17]. Genotypes 3 and 4 are found in industrialized countries including the United States, Europe, and Japan and are transmitted through ingestion of contaminated meat, usually pork, or blood transfusions [4, 14, 18–20].

HEV infection is considered as an emerging infectious disease with global concern [21]. Acute HEV infection accounts for a significant proportion of the acute hepatitis cases of unknown etiology [22, 23]. The global seroprevalence of antihepatitis E IgG in low-to-medium income countries such as Afghanistan and Iran as well as in industrialized countries, ranged from 2% to 68% [24–26]. Being male, veterinary and slaughterhouse workers, having pet animals or in close contact with animal reservoirs, especially swine, living in an endemic area, or consuming raw liver or internal organs meats were reported to be highly associated with positive HEV IgG antibodies [27, 28]. HEV is not an officially notifiable disease in Palestine (West Bank, Jerusalem, and Gaza Strip). Palestine is surrounded by HEV endemic countries and adjacent to the Israeli industrialized core which is home to immigrants from different countries such as European countries, the United States, and African countries including Sudan and Ethiopia. In addition, the possible link between HEV infection and animal reservoirs such as wild boars and the unknown seroprevalence of HEV and risk factors puts forward the aim to investigate the seroprevalence of HEV antibodies (IgG) and the associated risk factors.

## 2. Materials and Methods

### 2.1. Study Design and Population Size

**Figure 1:**
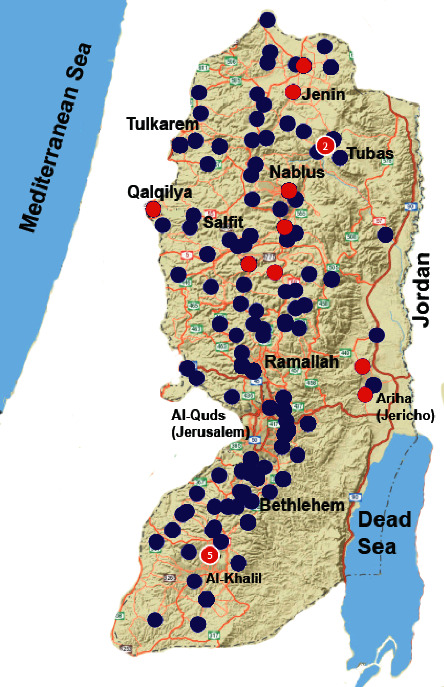
Spot map of the 16 HEV-IgG positive cases (red). The blue circles represent study participants from the 11 Palestinian districts. The numbers in the red circles represent the number of HEV-IgG positive samples in that geographical spot.

In the absence of any official data on the prevalence of HEV in Palestine, sample size was calculated based on the Daniel equation assuming a prevalence of 50% and a precision of 5%. According to the equation, the 50% assumption ensures the maximum peak of the sample size. The calculated sample size was 400; however, an extra number of samples (8%) were collected and tested to account for any drop out of cases. All individuals suffering from signs or symptoms of acute hepatitis, previously reported HBV and/or HCV positive, or immunocompromised individuals were excluded from the study. In this cross-sectional study, 432 healthy Palestinian individuals were selected from 40 localities (city, village, refugee camp, and Bedouin encampment) in the eleven districts of the West Bank and Jerusalem in Palestine during the period of March 2015 to March 2017. Participants were conveniently selected from individuals visiting the primary health care centers from all districts of the West Bank (Figure 1). Individuals with laboratory-confirmed positive results for hepatitis A, B, and C viruses were excluded.

### 2.2. Data and Sample Collection

All the participants were interviewed to collect information using a questionnaire. The interview included sociodemographic characteristics such as age, sex, educational level, and marital status and possible risk factors such as behavioral habits, eating habits, water source, contact with animals, blood transfusion, surgical procedures, travel history, and socioeconomic status such as income and occupation. Directly after the interview, five milliliters of whole blood samples were collected in plain tubes. The blood samples were centrifuged and the sera were separated and transferred into 1.5 mL microtubes and stored at −20°C until tested.

### 2.3. Laboratory Tests

All the sera were tested for HEV-IgG using an indirect ELISA (Fortress Diagnostics). All the samples that yielded HEV-IgG positive results were further tested for HEV-IgM using the direct ELISA method. The procedures of the two ELISA tests were based on the recommendation of the manufacturer's instructions. Both assays utilized recombinant HEV antigens, which are highly conserved among different HEV genotypes. Each sample was tested in duplicates. The results of both the HEV IgG and IgM were interpreted according to the manufacturer's instructions. The sensitivity and specificity of the assay kit were 99.5% and 99.6%, respectively.

### 2.4. Statistical Analysis

A database was set up for statistical analysis using the Epi Info software version 7.2.4.0 to build frequency tables and 2 × 2 contingency tables. Comparison of nominal and categorical variables between groups was assessed by using the chi-square test and Fisher's exact test. The level of significance was set to *P* < 0.05 at 95% confidence interval. HEV statistically significant spatial clusters were calculated and mapped using SaTScan version 9.7. The analysis was based on using the Poisson model to find low and high probability clusters in the study area. The Poisson model depended on the spatial coordinates, number of cases, and population size of that geographical location. The maximum spatial cluster size with statistical significance (*P* < 0.05) was defined as the circular window that has a radius of 5 km and contains a maximum of 25% of the population at risk.

### 2.5. Ethical Consideration

The study was approved by the Palestinian Ministry of Health under reference number 145/1541/2015. Verbal informed consent was obtained from all participants or the guardian in the case of minors. Patients' data were adequately anonymized.

## 3. Result

The overall prevalence of HEV-IgG in the study population was 3.7% (16/432). However, HEV-IgM was not detected in any of the 16 HEV-IgG positive samples indicating the absence of acute cases of infection. The study population consisted of an equal ratio of males and females. The highest number of participants was from the Al-Khalil district (*N* = 109), the most densely populated district in Palestine. Approximately two thirds of the participants were adults (Table 1).

The 16 HEV-IgG positive samples were spatially mapped by SaTScan v.9.7 with all participants mapped as a background. The purely spatial analysis did not detect any statistically significant cluster related to the distribution of HEV-IgG cases. Al-Khalil had 5 cases, but the number of participants from Al-Khalil was the highest (*N* = 109). However, the sample distribution in Figure 1 showed that the HEV-IgG positive cases followed the general pattern of distribution of the 432 participants, who are sporadically spread all over the West Bank.

Table 2 shows that HEV-IgG positive samples were predominantly male (69%) but statistically insignificant. In addition, the seroprevalence varied by age group but was statistically insignificant; children (0–19 years) and adults over 40 years had the highest prevalence (4.9–5.6%) than those in young adults (20–39 years). Moreover, the number of HEV-IgG samples was significantly higher in individuals who had low levels of education than those with higher levels of education (*P*=0.04). Also, living in the southern parts of the West Bank, in particular in Al-Khalil, significantly increases the risk of contracting HEV infection (*P*=0.018). Contrary to this, socioeconomic status (SES) including monthly income and occupation did not play any significant role in the circulation of HEV infection (*P*=0.42 and 0.48) (Table 3).

The clinical history of individuals including surgical intervention and blood transfusion was shown to have no significant effect on the HEV infection rate (Fisher's exact test, *P*=0.4). Similarly, factors affecting personal hygiene such as the source of drinking water, type of toilet used, toilet link, and contact with animals did not significantly affect the spread of HEV infection (Table 4).

## 4. Discussion

In the last couple of decades, HEV has been reported to be endemic in many developing and developed countries [14, 16, 18, 19, 22, 23, 28, 29]. However, the seroprevalence of hepatitis E showed considerable interregional and intraregional variation ranging from 1% in Iran to 49.8% in Bangladesh [30, 31]. The seroprevalence of HEV-IgG in the human population has been recognized as high (≥20.0%), medium (10.0–19.9%), and low (<10.0%) [32]. The low prevalence (3.7%) among the Palestinian population reported in the present study and the prevalence among the Jewish population (3.1%) are concordant due to close proximity between the populations. In another region such as southern Europe, the prevalence tends to be higher than in the Middle East such as Croatia (5.6%), Italy (4.3–5.38%), and Catalonia, Spain (7.3%) [33, 34]. On the contrary, high prevalence has been reported by others in Jordan (30.9%), among the Bedouin and non-Bedouin Palestinian Arabs in the Negeb desert (21.6% and 15.0%, respectively) and in Turkey (12.8%) [32, 35, 36]. The variation in the seroprevalence between the abovementioned studies including ours is partly due to the study population, geographic location, year of the study, sociological and economic status of the study population, dietary habits, and type of assay used. Eating habits based on religion such as consuming raw or undercooked pork meat have been suggested as one of the reasons of the high HEV prevalence [37], which partly explains the low prevalence among the Muslim and Jewish communities in our region.

The spatial analysis in the present study did not show any statistically significant clustering related to the distribution of HEV-IgG positive samples. The absence of any significant clusters can be due to the low number of HEV positive IgG participants. However, a significant number of HEV-IgG positive samples were reported in those participants living in the southern part of the West Bank (*P*=0.012). Approximately 25% (*n* = 102) of the sample size was from the Al-Khalil district; however, the population of this district is 24% of the total population in the West Bank (0.7/2.9 million), therefore, confirming the significance of the results [38]. Studies showed clear intercountry variation in seroprevalence of the HEV-IgG in Turkey and Iran ranging from 7.5 to 16% and 1.1% to 14.2%, respectively [30, 39]. The intercountry variation in seroprevalence including ours might be explained by differences in lifestyles, risk factors, levels of exposure, geographic regions, socioeconomic status, and hygienic behaviors.

In the present study, males had greater HEV prevalence than females, but insignificantly so, which is in complete congruence with other studies [40], while other studies reported contradictory results [33, 41]. However, these results should be interpreted with caution due to the small number of HEV IgG-seropositive samples in our study.

The association of HEV seropositivity with age was observed in our study, but the differences were not significant. However, categorizing age revealed that the young participants (<0–19 years) and adults over 40 years had higher HEV seropositivity compared to adults between 20 and 40 years old. Similar results were reported in Jordan, in which a high prevalence of HEV infections was reported in children less than 15 years of age and the older age group (>50 years) in comparison with other intermediate age groups. Several previous studies have also reported an association between HEV seropositivity and age; however, some did not include children [32, 33, 42]. The high prevalence of HEV in the young age group (0–19) might be explained by the outdoor exposures to infection, given their propensity to leave home more than the intermediate age groups. On the other hand, the high prevalence among the older age group is probably due to longer exposure to the virus over a lifetime. Other studies in both Switzerland and France did not find any significant association between age and HEV seropositivity [43, 44]. In consistence with previous studies, our study divulged that the level of education was inversely proportional to the HEV seropositivity [45]. High education levels can be reflected in good personal hygiene and proper health behaviors. On the other hand, conflicting results to this study were reported showing no difference in the seropositivity of HEV-IgG with regard to educational level [33, 46].

Socioeconomic status parameters such as income and occupation appear to be insignificantly associated with increased HEV seropositivity. Recent studies reported similar findings [33, 35, 47]. Using wells as a source of water and living in close proximity to animals were reported as risk factors of HEV infection [33, 48]. In the present study, HEV seropositivity among those who used wells as a source of water and lived in close contact with animals was higher, but the difference was not statistically significant. Similar result had been reported recently from Jordan. Again, the insignificant result might be explained by the low number of positive HEV IgG samples in our study.

Finally, our study did not find any significant association between HEV infection and surgical intervention and blood transfusion. Previous studies showed varying levels of significant increases in transfusion-transmitted cases of HEV infections from asymptomatic blood donors causing acute hepatitis, but in immunosuppressed and hemodialysis patients [3, 9–12, 49, 50].

The main limitations in the present study are the small number of positive samples, the inability to collect some demographic data such as the specific age after 40 years of age, and socioeconomic status parameters such as the specific amount of income of participants.

## 5. Conclusion

In conclusion, the overall seroprevalence of HEV in the general population of the West Bank, Palestine is low. Several well-investigated risk factors cannot be supported by our results due to the small number of positive HEV-IgG samples. Finally, this study is useful for providing a first look into the epidemiology of HEV in Palestine.

## Figures and Tables

**Table 1:** The general characteristics of the study population.

Characteristic	*N* (%)
Male: female ratio	211 : 217 (1 : 1)
Total	428
Age group	
Children (<18 yrs)	160
Adults (≥18 yrs)	271
Total	431
Districts	
Jenin	40
Tulkarem	19
Salfit	10
Qalqiliya	20
Tubas	10
Nablus	67
Ramallah	61
Ariha	10
Al-Quds	54
Bethlehem	32
Al-Khalil	109
Total	432

**Table 2:** Demographic and laboratory test results of the 16 HEV-IgG positive samples.

Code number	Sex	Age	Place of residence	Result of IgG	Result of IgM
Salf2	Male	3	Salfit city	Positive	Negative
Salf9	Male	50	Salfit (Qira town)	Positive	Negative
Jir9	Male	45	Ariha	Positive	Negative
Qal10	Female	15	Qalqailiya city	Positive	Negative
Tob2	Male	8	Tubas	Positive	Negative
Jen5	Female	35	Jenin-Qabatiya	Positive	Negative
Jen35	Male	9	Jenin	Positive	Negative
Ram35	Male	18	Ramallah-Jifna village	Positive	Negative
HC4	Male	42	Al-Khalil city	Positive	Negative
HC9	Female	68	Al-Khalil city	Positive	Negative
HC29	Male	15	Al-Khalil city	Positive	Negative
HC47	Male	3	Al-Khalil city	Positive	Negative
HYE3	Female	43	Al-Khalil-Yatta village	Positive	Negative
N33	Male	42	Nablus city	Positive	Negative
N54	Female	24	Nablus-Huwwara village	Positive	Negative
N77	Male	18	Nablus-Libban village	Positive	Negative

**Table 3:** Possible demographic and socioeconomic risk factors affecting the prevalence of the hepatitis E virus.

Variable	# HEV-positive (%)	# HEV-negative	*P*value^*∗*^
Seropositive	16 (3.7)	416	
Sex			
Male	11 (5.1)	202	0.1
Female	5 (2.2)	214	
Age group (y)			
0–9	4 (4.9)	77	0.12
10–19	4 (5.2)	73	
20–29	1 (1.1)	83	
30–39	1 (1.1)	83	
>40	6 (5.6)	100	
Residence			
Southern WB*∗∗*	5 (3.5)	136	0.012
Middle WB	3 (2.4)	123	
Northern WB	8 (4.8)	157	
Education level			
<Low school	15 (4.9)	288	0.04
>High school	1 (0.77)	128	
Income			
<$450	3 (5.7)	49	0.42
>$450	13 (3.4)	367	
Occupation			
Student	3 (3)	96	0.48
Housewife	4 (4.9)	77	
Worker	4 (4.3)	88	
Clerical worker	1(1.1)	80	
Health professional	0 (0)	27	
Others	4 (7.7)	48	
Travel aboard			
Yes	5 (3)	166	0.60
No	11 (4.4)	250	

^*∗*^ *X* ^2^ and Fisher's exact test; ^*∗∗*^WB, the West Bank.

**Table 4:** Clinical history and personal hygiene risk factors of HEV IgG positive participants.

Characteristic	# HEV-positive	# HEV-negative	*P* value^*∗*^
Drinking water			
Well	3	32	0.31
Bottle	0	0	
Pipe in house	13	354	
Toilet type			
Flush	10	279	0.58
Open pit	6	121	
Toilet link			
Septic tank	10	251	0.15
Sewage network	6	149	
Animal contact			
Yes	10	192	0.3
No	6	208	
Surgical intervention			
Yes	7	132	0.4
No	9	284	
Blood transfusion			
Yes	0	27	0.61
No	16	373	

^*∗*^ *X* ^2^ and Fisher's exact test.

## Data Availability

Data supporting this research are available from corresponding author on reasonable request.
